# An electrophysiological perspective on Parkinson’s disease: symptomatic pathogenesis and therapeutic approaches

**DOI:** 10.1186/s12929-021-00781-z

**Published:** 2021-12-09

**Authors:** Lan-Hsin Nancy Lee, Chen-Syuan Huang, Hsiang-Hao Chuang, Hsing-Jung Lai, Cheng-Kai Yang, Ya-Chin Yang, Chung-Chin Kuo

**Affiliations:** 1 Department of Physiology, National Taiwan University College of Medicine, 1 Jen-Ai Road, 1st Section, Taipei, 100 Taiwan; 2 Department of Neurology, Fu Jen Catholic University Hospital, New Taipei, Taiwan; 3 Department of Neurology, National Taiwan University Hospital, Taipei, Taiwan; 4 Graduate Institute of Biomedical Sciences, College of Medicine, Chang Gung University, Taoyuan, Taiwan; 5 National Taiwan University Hospital, Jin-Shan Branch, New Taipei, Taiwan; 6 Department of Biomedical Sciences, College of Medicine, Chang Gung University, 259 Wen-Hwa 1st Road, Kwei-Shan, Taoyuan, 333 Taiwan; 7 Neuroscience Research Center, Chang Gung Memorial Hospital, Linkou Medical Center, Taoyuan, Taiwan

**Keywords:** Basal ganglia circuitry, Subthalamic nucleus, Brain rhythms, Burst discharges, Cortico-subcortical re-entrant loops, Brain stimulation, Hyperdirect pathway, Hyperkinesia, Hypokinesia, Motor control

## Abstract

Parkinson’s disease (PD), or paralysis agitans, is a common neurodegenerative disease characterized by dopaminergic deprivation in the basal ganglia because of neuronal loss in the substantia nigra pars compacta. Clinically, PD apparently involves both hypokinetic (e.g. akinetic rigidity) and hyperkinetic (e.g. tremor/propulsion) symptoms. The symptomatic pathogenesis, however, has remained elusive. The recent success of deep brain stimulation (DBS) therapy applied to the subthalamic nucleus (STN) or the globus pallidus pars internus indicates that there are essential electrophysiological abnormalities in PD. Consistently, dopamine-deprived STN shows excessive burst discharges. This proves to be a central pathophysiological element causally linked to the locomotor deficits in PD, as maneuvers (such as DBS of different polarities) decreasing and increasing STN burst discharges would decrease and increase the locomotor deficits, respectively. STN bursts are not so autonomous but show a “relay” feature, requiring glutamatergic synaptic inputs from the motor cortex (MC) to develop. In PD, there is an increase in overall MC activities and the corticosubthalamic input is enhanced and contributory to excessive burst discharges in STN. The increase in MC activities may be relevant to the enhanced beta power in local field potentials (LFP) as well as the deranged motor programming at the cortical level in PD. Moreover, MC could not only drive erroneous STN bursts, but also be driven by STN discharges at specific LFP frequencies (~ 4 to 6 Hz) to produce coherent tremulous muscle contractions. In essence, PD may be viewed as a disorder with deranged rhythms in the cortico-subcortical re-entrant loops, manifestly including STN, the major component of the oscillating core, and MC, the origin of the final common descending motor pathways. The configurations of the deranged rhythms may play a determinant role in the symptomatic pathogenesis of PD, and provide insight into the mechanism underlying normal motor control. Therapeutic brain stimulation for PD and relevant disorders should be adaptively exercised with in-depth pathophysiological considerations for each individual patient, and aim at a final normalization of cortical discharge patterns for the best ameliorating effect on the locomotor and even non-motor symptoms.

## Background

### Parkinson’s disease (PD) as a disorder of deranged neural rhythms

PD is a common neurodegenerative disease. Clinically, PD is considered as a prototypical “hypokinetic” movement disorder because of the well-known symptoms of akinetic rigidity and bradykinesia. However, there are other cardinal symptoms of PD which are not apparently hypokinetic but carry hyperkinetic features, including tremor and propulsion. Pathologically, PD is usually characterized by α-synucleinopathy or α-synuclein accumulation in Lewy bodies, and consequent degeneration of the dopaminergic neurons in the substantia nigra pars compacta (SNpc) in the midbrain [1, 2]. Dopaminergic deprivation and subsequent dysfunction of the basal ganglia, the major projection targets of SNpc, presumably play a key role in the symptomatic pathogenesis of PD. The basal ganglia-thalamocortical circuits (cortico-subcortical re-entrant loops) travel from the cerebral cortex to the striatum, the globus pallidus (GP), the thalamus, and then back to the cerebral cortex, constituting the basic circuit loops underlying motor and many higher executive brain functions [3]. Conventionally, the cortico-subcortical re-entrant loops are subdivided into the direct and indirect pathways. The direct or striatonigral pathway travels via the striatum to the internal segment of GP or the substantia nigra pars reticulata (Gpi/SNpr). The indirect or striatopallidal pathway travels via the striatum to the external segment of GP (GPe), the subthalamic nucleus (STN), and then Gpi/SNpr [3–6]. As the major projection neurons in the striatum and GP are GABAergic and those in STN are glutamatergic, the striatonigral and striatopallidal pathways were conventionally viewed as overall excitatory and inhibitory inputs to the thalamocortical networks, respectively. Accordingly, hypokinetic movement disorders (e.g. PD) were hypothesized as consequences of abnormally decreased activities of striatonigral, and hyperkinetic disorders (e.g. chorea in Huntington’s disease) were ascribed to decreased activities of striatopallidal pathways. The expression of different subtypes of dopamine receptors presumably differentiates the striatal neurons involved in the two pathways [7–9]. However, these classical rationales could not satisfactorily explain the success of deep brain stimulation (DBS) of STN, or less frequently, GPi [10–12]. It is intriguing that local application of a pure physical maneuver, i.e. passing electric currents into STN, is sufficient to ameliorate the cardinal symptoms ascribable to the deficiency of a chemical substance (dopamine) supplied from a remote site (SNpc), especially considering that DBS on STN most likely would directly modulate the activities of STN without discrimination between neurons bearing different subtypes of dopamine receptors. Anatomically, STN does take a strategic position to modulate the information flow in both direct and indirect pathways, and is directly innervated by the motor cortex (MC) via the corticosubthalamic fibers or the “hyperdirect” pathway [13]. A revisit of the functional cooperation or the correlative relationship among the direct, indirect, and hyperdirect pathways from a STN-centered view may help to elucidate the physiological and pathophysiological operations of basal ganglia [14]. An in-depth exploration of STN activites in normal and PD subjects, either at baseline or with DBS, should also be fundamental. Although the symptomatic pathogenesis of PD is causally linked to deranged STN neural rhythms [14–19], the role of MC is also of great interest. As MC provides the most prominent and fastest direct excitatory drive to STN, MC could be very influential on STN activities. Moreover, MC assumes the most strategic coalescing point of the major (hyperdirect, direct, and indirect) pathways of the cortico-subcortical re-entrant loops, and is the origin of the final common descending pathways of motor commands. Perturbations of STN activities therefore could not only result from perturbations of MC activities directly, but also result in perturbations of MC activities via the re-entrant loops. This then gives rise to erroneous descending motor commands and consequent locomotor deficits in PD.

## Main text

### Two major modes of STN activities and the ionic currents underlying burst discharges

**Fig. 1:**
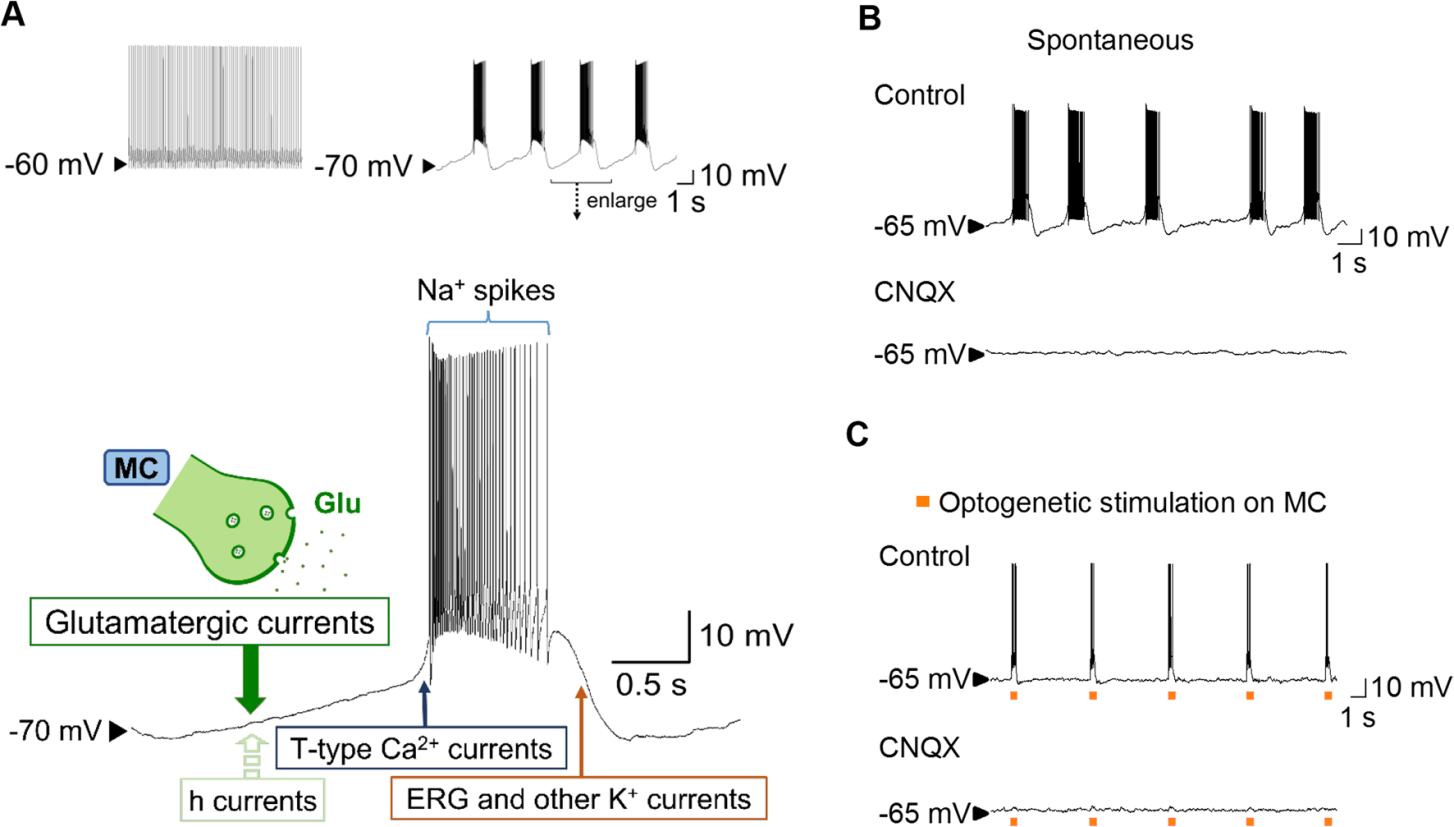
MC drive and major currents involved in the generation and shaping STN bursts. **A** Top, Membrane hyperpolarization (e.g. in a dopamine-deprived state) facilitates the burst mode of discharges in STN neurons. Bottom, A schematic drawing shows that intrinsic h currents in STN neurons could be insufficient to sustain autonomous burst discharges. The gradual depolarization phase in STN bursts very much relies on the glutamatergic currents provided by the input from the hyperdirect (corticosubthalamic) pathway. This endows STN bursts a strong relay feature for the MC-STN information flow. Once the membrane potential is depolarized enough to bring the T-type Ca^2+^ channels into a positive feedback activation cycle, the membrane potential is rapidly depolarized and positive-feedback activation of Na^+^ channels is involved to fire spikes. The burst plateau would sustain until enough “slow” K^+^ channels, like ERG channels, are opened to repolarize the membrane to a marked hyperpolarized level. **B** and **C** In-vitro slice recordings show that STN burst discharges are abolished by CNQX (20 μM) whether the bursts are spontaneous (part B, from a 68-day-old normal male Wistar rat) or evoked by optogenetic stimulation directly on the motor cortex (part C, from a 40-day-old *Thy1*-ChR2-EYFP transgenic C57BL/6 mouse). The generation of STN bursts thus is not so autonomous, and the glutamatergic input from MC seems to be an absolute requirement in most cases. Refer to Huang et al. 2021 [50] for details of experimental methods and analysis

An increase in burst activities in STN is a well-recognized electrophysiological marker of PD [20–22]. The STN neurons exhibit at least two distinct firing patterns, namely tonic and burst modes of discharges [23, 24]. In general, a neuron that fires in the tonic mode (or the spike mode) would respond to the input stimuli with changes in activities according to the spatiotemporal summation of the input signals. The tonic mode therefore is referred to as a relay mode for the major function of information conveyance. On the other hand, burst discharges could be self-repetitive or autonomous, repeating “by themselves” irrespective of the input signals [25, 26]. Burst discharges are thus conventionally viewed as a segregation mode that would interfere with or even interrupt information flow in the neural network, constituting the neurophysiological basis for temporary suspension of a specific part of neural networks such as the cases in sleep and selective attention [27–30]. When a neuron fires in repetitive burst discharges, it does not have a stable resting membrane potential. Instead, it is gradually depolarized by, in most known cases, the intrinsic hyperpolarization-activated currents (*I*_h_) or HCN channels [31, 32] after repolarization from the previous activities. The membrane becomes more rapidly depolarized when it reaches the threshold potential that drives the activation of T-type Ca^2+^ channels (T-channels) into a positive feedback cycle [14, 16, 33], and depolarizes the membrane to the threshold of firing Na^+^ spikes (i.e. activation of Na^+^ channels into a positive feedback cycle). The Na^+^ spikes are generated and so quickly repeated probably with the involvement of resurgent Na^+^ currents [34, 35] as to make a “burst” of discharges, which would be ended with adequate activation of slowly activated K^+^ channels and consequently membrane hyperpolarization to complete the cycle [17, 36–38, 40] (Fig. 1). The hyperpolarization phase also serves to recover inactivated T- and Na^+^ channels for the next cycle [39–41]. In fact, the switch between the spike and burst modes is critically dependent on the voltage of the hyperpolarization phase which determines the availability of T- and Na^+^ channels, and a difference by just a few mV could make the switch [28, 39, 42] (Fig. 1). In this regard, it is of note that the excessive STN bursts in PD are likely a direct effect of dopaminergic deprivation which tends to hyperpolarize STN neurons to facilitate the burst mode of discharges [23, 43–45].

### The novel “relay burst mode” of discharges in STN

As we have mentioned, the functional consequences of neuronal burst discharges were traditionally centered on interruption of neural information relay because of the autonomously repetitive feature of this discharge pattern. However, it is also plausible that the dense discharges in a burst may contribute to a strong and phasic delivery of neurotransmitters to the down-stream neurons, recruiting followers and thus the network into more synchronous activities [28, 46, 47]. The burst mode of discharges therefore may have a functionally more complicated sense related to information relay in addition to segregation. In this regard, we have shown that in STN and telencephalic structures such as amygdala, cell-intrinsic *I*_h_ currents are usually inadequate and extrinsic glutamatergic currents are necessary to bring up burst discharges and consequently seizure activities or dysrhythmias in the telencephalic circuitry or cortico-subcortical re-entrant loops [48–51]. Consistent with the proposed automaticity in STN neurons [34], we did find that a small proportion of STN neurons are capable of burst discharges in the presence of AMPA receptor antagonists [50]. However, the complete elimination of bursts by AMPA receptor antagonists in a large proportion of neurons, and the faithful induction of STN burst discharges by direct excitation of MC neurons or corticosubthalamic fibers strongly argues for the prevalence of a novel form of “relay burst” discharges in STN [50] (Fig. 1). Although GABAergic synaptic input could provide a hyperpolarizing force to activate *I*_h_ in rhythmogenic networks [15, 52–54], the role of a direct excitatory drive of burst discharges from the network (synaptic) level has not been well characterized. The demonstration of the crucial role of glutamatergic (particularly AMPA receptor-mediated) synaptic input for the generation of burst discharges not only provides a more comprehensive molecular view on burst genesis, but also lends a strong support for the relay sense of burst discharges in STN and probably also many other neurons. We have shown that the MC-triggered exaggerated burst activities in STN in PD animals could in turn trigger highly correlative electrophysiological activities in MC and then in skeletal muscles. This results in apparent hyperkinetic behaviors (e.g. tremors) which share very similar electrophysiological, behavioral, and therapeutic profiles to those in PD patients [50]. These findings argue for more positive actions rather than a pure negative action of burst discharges in STN (and probably also in the other structures of the cortico-subcortical re-entrant loops) on network synchronization and information relay (e.g. appropriating and synchronizing neural activities in specific parts of the networks). In other words, the functional implications of STN bursts in neural information relay may go well beyond the hyperdirect MC-STN pathway and contribute to the oscillating activities in the entire cortico-subcortical re-entrant loops.

### The causal role of excessive STN bursts in hypokinetic PD symptoms and the mechanism underlying DBS action on STN

Excessive STN burst discharges constitute a consistent and pathognomonic electrophysiological finding in PD [20, 22]. We have further established that excessive STN burst discharges are not just an associated phenomenon, but have a causal relation with parkinsonian locomotor deficits. Maneuvers which decrease the availability of T-channels, such as local application of T-channel blockers Ni^2+^ or mibefradil to STN, result in a decrease in STN bursts and amelioration of hypokinetic symptoms in parkinsonian animals [16]. Moreover, the effect of DBS on STN relies much more on the total charges delivered than the pulsatile pattern or frequency of stimulation [18]. For example, slight lengthening of the pulse width turns the ineffective low-frequency DBS into an effective one (although the pulse is given in the same low frequency). Even depolarizing DC currents which carry no pulsatile patterns make a very effective DBS protocol on the locomotor deficits in PD animals. Concurrent studies in brain slices show increasing membrane depolarization and T-channel inactivation in STN neurons with increasing amplitude of extracellularly applied depolarizing DC currents [14, 16, 18]. On the other hand, extracellularly applied hyperpolarizing DC currents increase the availability of T-channels and induce excessive STN bursts as well as hypokinetic locomotor symptoms in normal animals (with intact dopamine supply [16]), and even have a prominent ameliorating effect on animal models and a patient with hyperkinetic movement disorders [19]. Consistent with the idea that the K^+^ channels slowly activated during the plateau phase are crucial for burst termination, we found that ERG K^+^ channels are very influential on the repolarizing phase of STN burst plateau [17]. ERG channels open and are inactivated at the plateau phase of burst discharges. Once repolarization starts, the inactivated ERG channels are rapidly recovered via the open state but are slowly deactivated, giving rise to marked membrane hyperpolarization to facilitate the recovery of inactivated T- and Na^+^ channels for the next cycle. ERG channel inhibitors thus attenuate burst discharges in STN and ameliorate locomotor deficits in parkinsonian rats, whereas ERG channel activators promote burst discharges in STN and impair motor function in normal rats [17]. The ERG K^+^ channel, with its unique gating properties, has long been recognized for its strong modulatory effect on cardiac action potentials and rhythms. The channel is thus a major pharmacotherapeutic target for cardiac arrhythmias [55–58]. It is interesting that cardiac antiarrhythmics could potentially be brain antiarrhythmics in relevant clinical settings. These findings not only argue that excessive STN bursts have a direct causal relation with the hypokinetic symptoms of PD, but also indicate that DBS on STN chiefly works by depolarization of STN neurons and consequent inactivation of T-channels and/or Na^+^ channels. T-channels, ERG channels, or the other elements critically configuring STN bursts may constitute potential targets for pharmacotherapies of PD and relevant neurological disorders in the future.

### The deranged MC-STN functional connections in PD

**Fig. 2:**
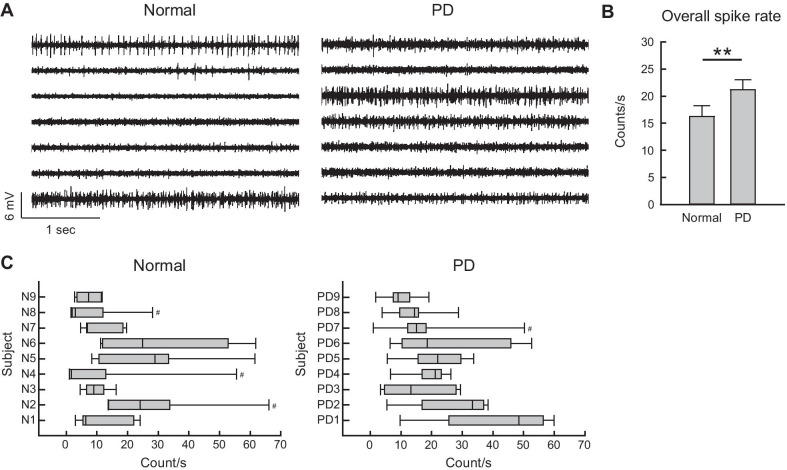
Higher MC activities in PD than in normal subjects. **A** Sample sweeps of MC spikes in a normal and a 6-OHDA hemiparkinsonian (PD) subjects (the left and right panels, respectively). Multi-unit (MU) recordings were obtained from arrays of seven separated insulated tungsten microwires placed in layer V-VI of MC in 8-week-old male Wistar rats. MC discharges at the seven recording sites (from top to bottom) are apparently more diffuse in PD, in comparison to the unevenly distributed pattern in normal subjects (see part C). **B** A 30-s period of recordings devoid of significant noise from the seven sites were selected for MU analysis for each subject. The sweeps were processed offline by a spike detection software (Sciworks 10.0, Datawave Technology), and the detection threshold was set at 4 times of the estimated median absolute deviation of signals. All detects were included as spikes in multi-unit profiles without further analytic treatment such as clustering. There are higher MU spike rates in PD than in normal subjects (n = 63, or 7 leads in 9 subjects for both groups, **P < 0.01 by Mann–Whitney U test). **C** Box plots displaying the lower and upper quartiles and the median show the MC spike rate distribution among the seven recording sites in each subject. The whiskers mark the minimum and maximum values of the dataset to display the range of outliers. There are more subjects with box plots skewed rightward in normal than PD subjects, suggesting that the spike rats are less evenly distributed among different leads in normal. ^#^subjects with outliers having individual spike rates > 1.96 standard deviation

**Fig. 3:**
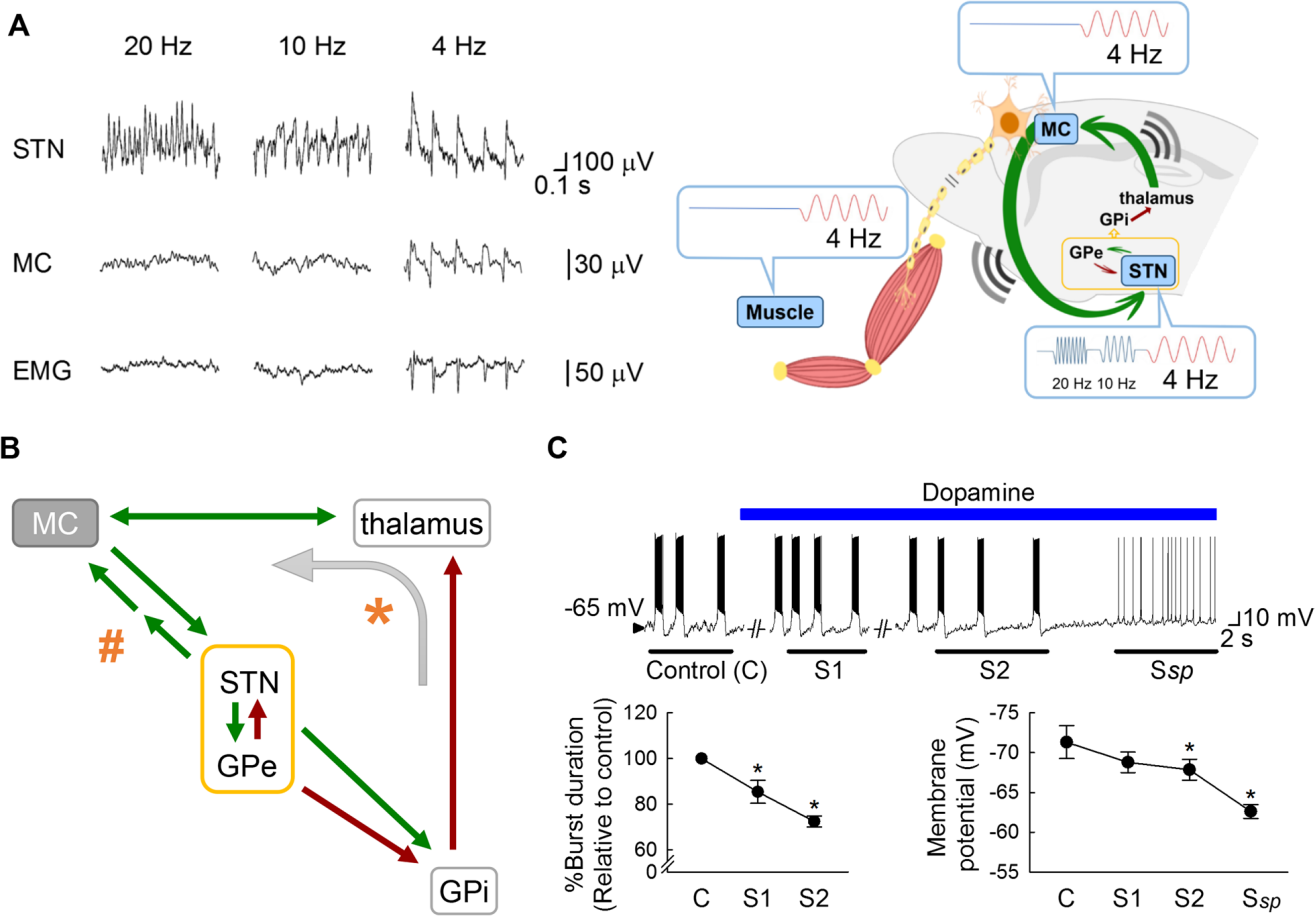
Information relay from STN to MC and the oscillating activities in the cortico-subcortical re-entrant loops. **A** With local application of 4-aminopyridine (4-AP), prominent repetitive burst discharges and oscillatory activities in LFP develop in STN at ~ 20, 10, or 4 Hz. However, coherent LFP oscillations in MC with vivid tremulous movement (muscle activities detected by electromyography or EMG) of the same frequencies could be discerned only with the ~ 4 Hz, but not ~ 10 or 20 Hz oscillating bursts (left, recording traces; right, a schematic drawing; also see [50]). This demonstrates that ~ 4 but not ~ 10 or 20 Hz is within the entrainable intrinsic frequency range of the major elements of the cortico-subcortical re-entrant loops, so that “resonance” of the major elements, including STN and MC, may ensue. Recordings were from an anesthetized 2-month-old parkinsonian (6-OHDA-lesioned) male Wistar rat. **B** A schematic drawing for possible major connections between MC and STN (green arrows: glutamatergic pathways; crimson arrows: GABAergic pathways). In contrast to the hyperdirect (corticosubthalamic) pathway which conveys MC drives directly to STN, there is no well-established direct input from STN to MC [123]. There are, then, two possible ways for the relay of information from STN to MC (as in part A). Indirect excitatory pathways via, for example, the pedunculopontine nucleus which has reciprocal connections with both MC and STN [124] and is also a locomotor center for the control of muscle tone [125, 126], may exist to relay the STN bursts back to MC with a phase lag (indicated by # and the two consecutive arrows). Together with the hyperdirect pathway, this may contribute to the necessary extrinsic excitatory drive for continual oscillations in the STN-GPe core. On the other hand, the STN-GPe oscillating activities may travel via GPi (indicated by * and the curve arrow), the main output station of basal ganglia, to implement rhythms to the thalamocortical oscillation circuitry. **C** In acute brain slices from parkinsonian (6-OHDA-lesioned) male Wistar rats (aged 5–6 weeks, n = 4–5), perfusion of dopamine (25 μM) gradually depolarizes the membrane and shortens the burst discharges in STN neurons until a stable spike mode of discharges (S*sp*) appears. The lowest membrane potential and the burst duration is measured and averaged from the three consecutive bursts immediately after the 30th sec of dopamine perfusion (S1) and just before the transition to the spike mode (S2). Data are mean ± SEM. *P < 0.05 compared to control (before dopamine), Friedman tests followed by Wilcoxon signed-rank tests for further pairwise comparison. See Huang et al., 2021 [50] for more experimental details in parts **A** and **C**

We have argued that excessive STN bursts have a direct causal relation with the locomotor deficits in PD, and that the STN bursts are in essence relay bursts driven by the glutamatergic input from MC (via the hyperdirect pathway). The symptomatic pathogenesis of PD therefore may also involve deranged MC activities or deranged STN responses to MC activities, or both. MC is also a target of dopaminergic innervation from SNpc [59]. MC activities therefore could also be altered in PD. There are, however, inconsistent reports on MC activities, such as changes in mean firing rates or burst discharges, and/or time-varying tuning of MC discharges during movement, after experimental dopaminergic deprivation [60–62] (for review see [63]). We also found there are subtle but definite changes in MC activities in animals with 6-hydroxydopamine-induced lesions in SNpc (Fig. 2). In terms of MC-STN transmission, there were proposals on the reduced MC innervation onto STN neurons in animal models of PD in several recent studies [64–66]. However, the findings are primarily anatomical, and functional studies are relatively deficient. On the other hand, there were quite a few reports arguing for enhanced functional MC-STN connections in PD, including human fMRI data [67–73]. We have characterized the enhanced functional connections in detail, and shown that the burst discharges in STN are much more increased at 0–150 ms after stimulation of the premotor cortex in PD (dopamine-deprived) than in normal animals [67]. Moreover, the STN discharges are accrued at ~ 9 ms after cortical stimulation in PD animals, whereas in normal ones the STN discharges are scattered in a much wider time range, with two small peaks at ~ 20 and ~ 60 ms [67]. In addition, MC stimulation-induced local field potential (LFP) signals in STN are stronger in PD [50]. With repetitive MC stimulation mimicking cortical activities during movements [74], each pulse faithfully generates a LFP response which is larger and reaches the maximum faster in PD than in normal rats [50]. These findings suggest that even though the corticosubthalamic fibers may be anatomically decreased, the MC-STN functional connection is “enhanced”, and probably more synchronized, in PD. Moreover, the altered MC-STN information relay may carry imperative behavioral and therapeutic implications. Local application of glutamatergic antagonists into STN would suppress STN bursts and ameliorate locomotor symptoms in PD animals [50, 67, 75, 76]. Although high-frequency electrical stimulation of the corticosubthalamic fibers may ameliorate parkinsonian hypokinetic symptoms [68] (presumably ascribable to a depolarizing effect like that of DBS on STN), optogenetic overpacings of the same fibers induce vivid parkinsonian locomotor deficits in normal animals [67]. In this regard, it is interesting to note that the MC-triggered exaggerated STN bursts could in turn trigger highly correlative activities in MC in PD animals with local application of A-type K^+^ channel inhibitors to STN (to increase cortico-subthalamic AMPA receptor-mediated fast glutamatergic synaptic transmission at both pre- and post-synaptic loci) [50] (Fig. 3). This vividly demonstrates that cortical activities may be further deranged via deranged subcortical elements in the re-entrant loops. The symptomatic pathogenesis of PD therefore is directly ascribable to excessive STN bursts, which are in essence relay bursts. It is in turn ascribable to both deranged MC activities and deranged STN responses to the deranged cortical drives.

### The network basis translating excessive STN bursts into locomotor deficits in PD

In view of the apparent direct causal relation, it would be interesting to decipher how excessive STN bursts shall lead to the locomotor deficits in PD. We have noted that deranged STN activities could in turn affect the operation of MC, the final common pathway of descending motor commands. Also, STN bursts are essentially relay bursts which are not intrinsically self-repetitive but rely on the extrinsic hyperdirect corticosubthalamic glutamatergic input to develop. Cues or intention of a movement, for instance, may increase corticosubthalamic glutamatergic transmission and thus cause a transient increase in STN burst discharges. STN bursts with densely repetitive spikes conceivably shall have a stronger inhibitory effect than isolated spikes on corticothalamic activities via GPi, probably serving as a “brake” on premature actions. This proposal explains the clinical findings that hemiballisms may develop with destructive lesions of contralateral STN. STN bursts could also provide strong glutamatergic drive on the reciprocally innervated GABAergic GPe. The STN-GPe “core pacemaker” of the cortico-subcortical re-entrant loops [15] is then subject to an escalated excitatory arm. The STN-GPe oscillations could be triggered and enhanced with STN bursts, exporting a stronger modulatory effect on the other structures in the cortico-subcortical re-entrant loops (Fig. 3). STN and GPe neurons were found to fire bursts of action potentials at early-stage sleep, at frequencies similar to coincident cortical slow-wave (~ 1 Hz) and spindle activities (~ roughly in the alpha range or ~ 7 to 14 Hz) [77]. The very wide frequency range supports a nature of relay bursts in STN and presumably the versatile effect of the glutamatergic input from MC. For example, strong cortical activation by sensory input may lead to a reduction of slow wave activities as well as a shift of STN–GPe bursts to tonic firings [77]. In vivo and in vitro findings further suggest that the STN–GPe network can support network oscillations for the generation of resting tremor [15, 50, 78]. Anatomically, GPi serves as the coalescing or nodal point of both STN and GPe outputs. Physiologically, this could be viewed as a fundamental design for the assured transmission of the STN-GPe oscillating burst activities to GPi to fulfill the function as a brake on the premature MC activities or “noises”. Consistently, parkinsonian monkeys with MPTP (1-methyl-4-phenyl-1,2,3,6-tetrahydropyridine) lesions show an increase in burst activities in not only GPe but also GPi [79–83]. The erroneous STN-GPe and then GPi oscillating activities may implement erroneous rhythms to thalamocortical activities, and thus pathognomonic rhythms in the cortico-subcortical re-entrant loops in PD (Fig. 3). For example, there could be highly concordant 4–6 Hz or high-frequency oscillating neuronal discharges in STN and GPi in PD patients, especially when there is vivid tremor [84–86]. The STN relay bursts, driven by MC, may entrain the STN-GPe core and then the entire re-entrant loops into coherent oscillating activities [50, 87]. MC activities are transmitted downstream to spinal cord and to STN in parallel. STN bursts shall not just loop back to MC to inhibit the ongoing activities, but also create an activity pattern to compensate precisely for the actions of the MC activities transmitting downstream during the looping time to complete the brake function. This is a relatively neglected part of the basal ganglia function. It could be the key to the intriguing coexistence of both hypokinetic and hyperkinetic manifestations of erroneous brain rhythms in PD (see below).

### The modulated hyperkinesia (tremor) on top of hypokinesia in PD and motor execution

**Fig. 4:**
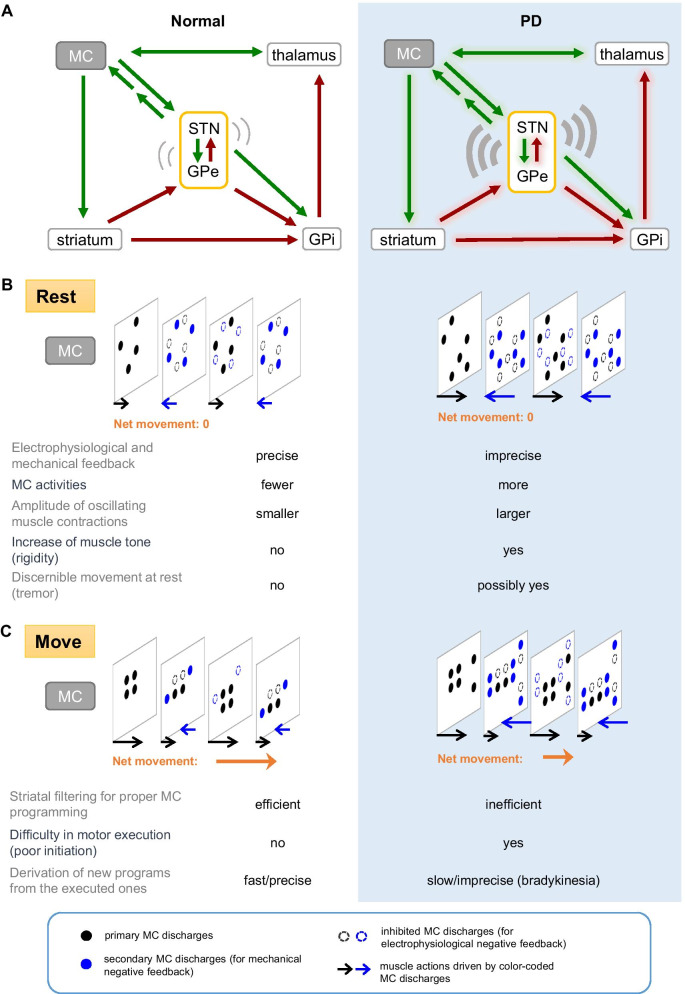
A functional view of the basal ganglia circuitry in normal (left column) and parkinsonian (right shaded column) motor control. **A** The cortico-subcortical re-entrant loops with delicately controlled oscillating activities in the normal state (left) or deranged oscillating activities in PD (right). MC discharges are transmitted to STN via the hyperdirect pathway, and then loop back to MC to have a negative feedback effect via the GABAergic GPi (i.e. MC-STN-GPi-thalamus-MC, green arrows: glutamatergic, crimson arrows: GABAergic). MC activities are also “filtered” by the striatum, and transformed chiefly as an inhibitory input to GPi via the direct pathway to modify the excitatory drive from STN. **B** The cortical process at rest. In the normal state (left), the original MC activities (black circles) are erased by the foregoing effect via GPi and conclude the electrophysiological negative feedback process (dashed black circles). However, activities of the lower motor neurons and muscle contractions may well be elicited by the downwardly transmitted MC discharges during the looping process. New (compensatory) MC activities (blue circles) therefore must also be generated for counteractions at the muscle level (the “mechanical” negative feedback) presumably via some indirect excitatory pathways from STN to MC. The mechanical negative feedback ensures no discernible “microscopic” movement at rest. These compensatory MC activities subsequently arouse similar responses, including the electrophysiological negative feedback via GPi (dashed blue circles) and the mechanical negative feedback via STN (the “resurgent” black circles). With timely and appropriately modulated STN bursts by phasic dopamine release, the primary and the compensatory MC activities are accurately configured. Meanwhile, the invisible microscopic movement or “microtremors” may constitute a major base for the genesis of muscle tone. The negative electrophysiological feedback is less precise in PD (right), with excessive STN bursts in response to corticosubthalamic input. Together with the presumable derangement in lateral inhibition in the striatum with dopamine deprivation, MC activities are increased. The mechanical feedback then is less precise, resulting in larger-scale alternating muscle contractions and thus cogwheel rigidity. Discernible tremor may develop, especially if there are enough residual dopaminergic neurons that the erroneously modified MC activities could be partly executed. Under such circumstances, 4–6 Hz could be the “best” oscillating frequencies for generation of manifest tremor, considering the potential phase lag between the coherent MC and STN oscillations. Tremor could be delicately modulated by changes in dopamine (or the other neuromodulators) levels and STN discharge patterns. Thus, psychomotor stress or an intention to move could enhance tremor which could then be suddenly abolished by relief of the stress or execution of the movement. **C** The cortical process on move. If relevant MC activities are continually presented (e.g. with a will of motion) to the striatum, the MC activity patterns would be gradually specified with striatal filtering in the normal state (left). Meanwhile, the STN drive on GPi is gradually weakened by the inhibitory effect via the direct pathway and/or the decreasing burst discharges with locally accumulated dopamine. Once the erasing actions are no longer complete, discernible movement would happen. This defines the “motor threshold”, which denotes the level for a discernible movement rather than a point of no return, as an executed pattern can still be modified any time. Discernible movement may by itself contribute to the making of the next MC activity patterns with rationales similar to the foregoing electrophysiological and mechanical negative feedbacks (e.g. sequential motions or “motor habits”). The sequential or repetitive moves in a movement set thus are easier to proceed once the first one is executed. In this case, the indirect pathway presumably assumes a more prominent role for the follow-up negative feedback to erase the executed MC activities because of weakened role of STN. In PD (right), initiation of a new motor task is difficult as repeated trials can hardly accumulate enough local dopamine and enough specified MC activities (e.g. with defective striatal filtering) for motor execution. However, if discernible motion does happen in this case, the motor threshold must have been surpassed and enough weakening of the electrophysiological and mechanical negative feedbacks achieved. The next move of the similar kind in a sequential set would then be easier. In general, the net movement of each sequential move would be less efficient and thus “bradykinetic” in PD because of the erroneous cortical programming and thus less efficient derivation of an appropriate pattern from the executed actions and counteractions. However, if the errors in programming happen to be in the same direction, cumulative temporal and spatial changes in motor scale may prevail with motor execution. This could be especially discernible with simple repetitive moves (e.g. micrographia or propulsive gait in clinical settings)

PD is not just a hypokinetic disorder characterized by “akinetic rigidity”. It is also an apparent hyperkinetic disorder for the prevalence of rest tremor typically before the late stage of the disease [88, 89]. Parkinsonian tremor is intriguing not only for the hyperkinetic features on top of vivid hypokinesia, but also for the evident modulation in its amplitude and frequency according to the psychomotor status (e.g. from an intention to the actual execution of a move, or from stressful to non-stressful situations). We have shown that the highly coherent burst discharges in the MC-STN axis could lead to muscle contractions and hyperkinetic behaviors mimicking tremor when the burst discharges are repeated at the theta frequencies (~ 4 to 6 Hz [50]; Fig. 3), roughly consistent with the frequency of tremor in PD patients [86]. Although reverberating discharges at a wider range of frequencies may be present in the STN-GPe oscillation core and the other key elements in the re-entrant loops, it seems that only a narrow frequency band or roughly theta oscillation-entrained MC discharges could have an evident behavioral penetrance to make discernible oscillating muscle contractions with a background condition of partial dopaminergic deprivation. Accordingly, block of corticosubthalamic glutamatergic transmission is sufficient to abolish STN bursts as well as oscillations in MC and ameliorate tremor-like hyperkinesia in PD animals [50]. In theory, the extrinsic excitatory input from MC to STN does not have to be burst discharges, but the spatiotemporal configuration of the input currents must play an imperative role in shaping the STN-GPe oscillations. For example, if MC and STN discharges could be so coherent at specific frequency bands that the MC drives timely arrive at the nadir phase of STN-GPe oscillating activities, the oscillations in the entire re-entrant loops will be “propelled” to continue or even enhanced. In this regard, manifest parkinsonian tremor may emerge within a specific oscillation frequency band because the phase lag between the coherent MC and STN oscillations could just fit the foregoing conditions for the optimal enhancement and execution of the descending motor commands (Fig. 3). Midbrain dopaminergic (e.g. SNpc) neurons also show tonic and burst discharges, and the switch to burst discharges is dependent on the glutamatergic innervations from STN and pedunculopontine nuclei [90–93]. Dopamine is especially triggered to release and may then be accumulated with repeated burst discharges of SNpc neurons, reaching a local concentration much higher (e.g. even to the millimolar range) than the tonic level with silencing of the striatal cholinergic interneurons [90]. The continuing intention of a move presumably is represented by sequentially modified activity patterns in MC, with the corresponding STN bursts gradually decreased or shortened by the locally accumulating dopamine like the scenario in Fig. 3. The move would actually happen (i.e. be executed) when the MC activity pattern, STN discharges, and local dopamine concentrations collaboratively reach appropriate levels. As the amount of dopamine may determine the prevalence and configuration of the bursts, and consequently the wavelengths (or frequencies) as well as band power of the LFP oscillations in STN and re-entrant loops, the presence of tremor in PD could be correlated with the residual dopaminergic supply (and thus simplistic supplement of dopamine may ameliorate tremor). The parkinsonian tremor in PD therefore is a kind of “rest tremor” because it is mainly discernible at rest and tends to disappear upon motor execution (e.g. when there is sufficiently high local dopamine to abolish STN bursts). However, it could actually be more manifest at the pre-action than the “true” rest conditions. The pre-action accentuation of PD tremor also lends a strong support for the idea that essentially all MC activities are transmitted to spinal cord for muscle actions (see Fig. 4). However, tremor also tends to disappear and is replacement by more prominent akinetic rigidity toward the late stage of PD when the residual dopamine is too low to sustain coherent oscillating discharges in the MC-STN axis and the re-entrant loops at optimal frequencies for the behavioral manifestations at the muscle level.

### Possible pathophysiological implications of beta synchronization in PD

In addition to excessive STN burst discharges, the other well reported electrophysiological abnormalities in PD are augmented beta power (“beta augmentation”) and enhanced synchronization between the beta oscillations in MC and STN (“beta synchronization”) [94–96]. The power of beta-band (~ 20 to 40 Hz) oscillations is enhanced in the LFP in MC and STN, and even in the other major sites in the cortico-subcortical re-entrant loops in PD [94, 97–100]. Beta augmentation has long been considered also as a critical element responsible for the locomotor deficits of PD. However, the idea is chiefly based on association studies, such as the correlation between severity of rigidity/bradykinesia and beta power/coherence, or the correlation between the efficacy of PD treatment and changes in beta power [101–107]. As a comparison, excessive STN burst discharges seem to be a rather consistent finding in PD. Although local injection of glutamatergic antagonists into STN would suppress both excessive STN bursts and beta augmentation in STN/MC, with improvement in locomotor activities in parkinsonian animals [67], beta synchronization may not be changed significantly with DBS therapy [108] (but see [109]). In addition, although MC-STN beta power could be increased even in early PD [110], beta augmentation is in general not as consistently present and could be absent in subjects with severe locomotor symptoms [111–114]. LFP reflects spatiotemporal changes of local currents, and is thus closely related to the multi-unit activities of regional neuronal ensembles. It is difficult to correlate beta (LFP) augmentation directly to the characters of the excessive STN burst discharges, which are in general a few hundred milliseconds or longer in length as well as repeated at a frequency of 1 Hz or lower [16]. At the cortical level, although corticofugal or pyramidal tract-type neurons were reported to have reduced activation with dopaminergic deprivation [60, 115], we found a general increase in multi-unit MC activities in parkinsonian animals (Fig. 2). There is also an increase in fMRI signals in MC in PD patients [116, 117]. The interrelations between multi-unit activity increases and beta augmentation await further exploration, but both may indicate derangements in motor programming at the cortical level (see also [118] and [119]). It is plausible that beta augmentation is related to some specific and delicate motor dysfunctions in PD, rather than a general marker for locomotor deficits.

## Conclusion

### A concluding synthesis: the distinctions between normal and parkinsonian motor control

All MC activity patterns are processed by the cortico-subcortical re-entrant loops and continually modified (Fig. 4). Meanwhile, MC activities are transmitted downwards for motor execution in parallel. MC activities could be rapidly “erased” by the hyperdirect pathway-driven STN discharges via GPi. The process, although rapid, may still take a few tens of milliseconds to complete in normal conditions [120]. The minimally “executed” MC activities during this looping time therefore necessitate counter-actions at the end-organ (e.g. muscle) level to be offset, so that the system returns to the original state. MC activities for counter-actions presumably are generated via a very rapid feedback from STN to MC to assure no discernible movement at normal rest. Indirect excitatory pathways from STN to MC similar to those described in Fig. 3 may sustain such a timely counteraction (Fig. 4B). This is probably also the reason why positive evoked potentials with a latency of just ~ 10 to 20 ms could be elicited by STN stimulation [121, 122]. Because the cortical events for counteractions would in principle also induce counter-counteractions, there could be reverberating minimum agonist and antagonist muscle contractions at rest, constituting the basis of muscle tone. MC activities are also “filtered” by the striatum before being relayed to GPi, where a thwarting effect of the direct pathway on the hyperdirect pathway may contribute to set the magnitude of the aforementioned reverberations. On the other hand, if continuous MC drives are generated with the desire of a specific motion, there are presumably more “mature” MC activity patterns after repeated cortico-subcortical re-entrant looping and striatal filtering (Fig. 4C). STN counteractions could then be so weakened by the well modified MC activities and locally accumulated dopamine that manifest motor actions ensue. In PD, the excessive STN bursts without timely and sufficient local accumulation of dopamine result in excessive suppression of motor execution. The impaired filtering function of dopamine-deprived striatum also leads to a weakened thwarting effect on STN counteractions at GPi and thus a tendency of over-counteractions and consequent larger oscillating activities in PD. MC activities therefore are increased, but are noisier and less efficient for the formation and execution of appropriate motor programs (Fig. 4). Therapeutic brain stimulation should then be exercised with in-depth pathophysiological considerations for each individual patient, aiming at normalization of MC discharge patterns for the best ameliorating effect on the locomotor and probably also the non-motor symptoms in PD.

## Data Availability

Not applicable.

## Abbreviations

6-OHDA: 6-Hydroxydopamine hydrobromide
4-AP: 4-Aminopyridine
AMPA: α-Amino-3-hydroxy-5-methyl-4-isoxazolepropionic acid
DBS: Deep brain stimulation
DC: Direct current
ERG K^+^ channel: Human ether-à-go-go-related K^+^ channel
fMRI: Functional magnetic resonance imaging
GABA: γ-Aminobutyric acid
GP: Globus pallidus
GPe: Globus pallidus pars externus (external segment of globus pallidus)
GPi: Globus pallidus pars internus (internal segment of globus pallidus)
HCN channel: Hyperpolarization-activated cyclic nucleotide–gated channel
LFP: Local field potentials
MC: Motor cortex
MPTP: 1-Methyl-4-phenyl 1,2,3,6-tetrahydropyridine
MU: Multi-unit
PD: Parkinson’s disease
SNpc: Substantia nigra pars compacta
SNpr: Substantia nigra pars reticulate
STN: Subthalamic nucleus
